# Allogeneic stem cell transplantation for mantle cell lymphoma—update of the prospective trials of the East German Study Group Hematology/Oncology (OSHO#60 and #74)

**DOI:** 10.1007/s00277-021-04506-y

**Published:** 2021-04-08

**Authors:** William H. Krüger, Carsten Hirt, Nadezda Basara, Herbert G. Sayer, Gerhard Behre, Thomas Fischer, Norbert Grobe, Georg Maschmeyer, Thomas Neumann, Laila Schneidewind, Dietger Niederwieser, Gottfried Dölken, Christian A. Schmidt

**Affiliations:** 1 Department of Internal Medicine C – Haematology and Oncology, Stem Cell Transplantation, Palliative Care – University of Greifswald, Ferdinand-Sauerbruch-Straße, 17475 Greifswald, Germany; 2 Department Haematology/Oncology, University Hospital Leipzig, Leipzig, Germany; 3 Department for Haematology and Oncology, Clinic for Internal Medicine II, University Hospital Jena, Jena, Germany; 4 Department Haematology/Oncology, Martin Luther University, Halle/S, Germany; 5 Department Haematology/Oncology, Otto von Guericke University, Magdeburg, Germany; 6 Dietrich Bonhoeffer Hospital, Neubrandenburg, Germany; 7 Hospital Ernst von Bergmann, Potsdam, Germany

**Keywords:** Mantle cell lymphoma, Allogeneic stem cell transplantation, Graft-versus-lymphoma effect, Clinical trial

## Abstract

Mantle cell lymphoma (MCL) is a non-Hodgkin’s lymphoma with an often aggressive course, incurable by chemotherapy. Consolidation with high-dose therapy and autologous stem cell transplantation (autoSCT) has a low transplant-related mortality but does not lead to a survival plateau. Allogeneic stem cell transplantation (alloSCT) is associated with a higher early mortality, but can cure MCL. To investigate alloSCT for therapy of MCL, we conducted two prospective trials for de novo MCL (OSHO#74) and for relapsed or refractory MCL (OSHO#60). Fifteen and 24 patients were recruited, respectively. Induction was mainly R-DHAP alternating with R-CHOP. Conditioning was either Busulfan/Cyclophosphamide or Treosulfan/Fludarabin. Either HLA-identical siblings or matched-unrelated donors with not more than one mismatch were allowed. ATG was mandatory in mismatched or unrelated transplantation. Progression-free survival (PFS) was 62% and overall survival (OS) was 68% after 16.5-year follow-up. Significant differences in PFS and OS between both trials were not observed. Patients below 56 years and patients after myeloablative conditioning had a better outcome compared to patients of the corresponding groups. Nine patients have died between day +8 and 5.9 years after SCT. Data from 7 long-term surviving patients showed an excellent Quality-of-life (QoL) after alloSCT. AlloSCT for MCL delivers excellent long-term survival data. The early mortality is higher than after autoSCT; however, the survival curves after alloSCT indicate the curative potential of this therapy. AlloSCT is a standard of care for all feasible patients with refractory or relapsed MCL and should offer to selected patients with de novo MCL and a poor risk profile. For defining the position of alloSCT in the therapeutic algorithm of MCL therapy, a randomized comparison of autoSCT and alloSCT is mandatory.

## Introduction

The introduction of rituximab, cis-platinum, and high-dose Ara-C into therapy protocols for mantle cell lymphoma (MCL) is landmark in the improvement of outcome and prognosis of these patients [1–3]. High-dose therapy (HDT) followed by autologous stem cell transplantation (autoSCT) as consolidation therapy has been broadly investigated for therapy of MCL. The experimental arm of the “E-MCL younger trial” represents an accepted standard of care for eligible patients with de novo MCL [3]. However, the survival curves show only a significant benefit in PFS for the intensified arm. Differences in overall survival are non-significant and these curves do not suggest a curative potential of the EMCL-younger protocol [3]. The presence of minimal residual disease prior and after autoSCT worsens the prognosis of patients suffering from MCL [4, 5]. To address this problem, rituximab maintenance therapy after autoSCT has been investigated with very promising results [6, 7].

Results of HDT+autoSCT in non-first remission are inferior to those obtained in the first remission [8, 9].

In contrast, the curative potential of allogeneic stem cell transplantation (alloSCT) in MCL, even in higher remissions, has been shown by several investigators [10–14]. However, alloSCT is associated with higher transplant-related morbidity and mortality than HDT+autoSCT. AlloSCT is the therapy of choice for eligible patients in higher remissions but the use of HDT+autoSCT as consolidation therapy in the first remission is discussed controversially [8]. A new potentially curative approach is the therapy of relapsed and refractory MCL with specific CAR-T-cells (KTE-X19). Wang et al. reported here excellent results after a follow-up of nearly 3 years [15].

A variety of retrospective analyses about alloSCT in MCL have been published; however, prospective trials are rare. Maris et al. pooled data from MCL-patients treated in different prospective trials and published excellent data for alloSCT after minimal conditioning [13]. The East German Study Group for Haematology and Oncology (OSHO) has conducted two prospective trials for patients with de novo MCL (OSHO#74) and for patients with the refractory or relapsed disease (OSHO#60). A total of 33 patients was transplanted in both trials with promising results [16]. Here the 2019 update of both trials is presented.

## Patients, material, and methods

### Patients, trials, and therapy

Patients, trials, and therapy have been described in detail previously [16]. Briefly, the trial OSHO#60 was open for patients with relapsed or refractory disease. Conditioning was treosulfan (3*12g/m^2^) + fludarabine (150mg/m^2^) ± rituximab 375mg/m^2^. Fifteen patients (male: 13, female 2, median age 61, range 45–65 years) were recruited into this trial. The patients were heavily pre-treated with a median of eight (range 6–13) cycles of chemotherapy from two (median, range 1–3) different regimens. No patient had a history of high-dose therapy followed by autologous stem cell transplantation. Primary therapy was classical chemotherapy for indolent lymphoma, partially in conjunction with rituximab. Other targeted drugs were unavailable or not established at that time. Re-induction prior to allogeneic SCT was mainly R-DHAP; other chemotherapy in conjunction with Rituximab was allowed. Three of these fifteen patients could not proceed to alloSCT because of progressive disease under salvage therapy (*n*=2) or the lack of a compatible donor.

OSHO#74 was open for patients with de novo MCL (AnnArbor ≥II). Induction therapy was by randomization either 6*R-CHOP or 3*R-CHOP/R-DHAP as introduced by the EMCL-network [3]. Twenty-four patients (male: *n*=18, female: *n*=6, median age 59 years, range 33–69 years) were included. For proceeding to alloSCT, at least a partial remission had to be reached. The age limit was 18–65 years, upper limit biologically. Related sibling donors or matched unrelated donors carrying ≤1 mismatch were allowed. GvHD-prophylaxis consisted of cyclosporine-A (CsA) and short-course methotrexate (MTX). In vivo T-cell depletion by antithymocyte globulin (ATG) was mandatory in case of mismatched or unrelated transplantation [16].

Patient details are shown in Table 1.

**Table 1:** Patients details

Parameter	Salvage SCT (OSHO #060)	Primary SCT (OSHO #074)	Total
	*N* (% or range)	*N* (% or range)	*N* (% or range)
Overall recruitment			
Patients recruited	15	24	39
Male/female	13/2 (87/13)	18/6 (75/25)	31/8 (79/21)
Patients allografted/dropouts	12/3 (80/20)	21/3 (88/12)	33/6 (85/15)
Reasons for dropout	-Progressive disease (*n*=2) -No stem cell donor	-Diagnosis revised -Patient refused allo-SCT -Physician’s discretion	6
Patients undergoing allogeneic stem cell transplantation			
Male/female	11/1 (92/8)	15/6 (71/29)	26/7 (79/21)
Age at SCT	61 (45–65)	59 (33–69)	59 (33–69)
MIPI	n.a.	Low risk: 7 (33) Intermediate risk: 4 (19) High risk: 6 (29) Missing: 4 (19) Median (range): 5 (2–9)	n.a.
Pre-treatment		n. a.	n.a.
Cycles of chemotherapy	8 (6–13)		
Regimen of chemotherapy	2 (1–3)*		
Disease status at study inclusion	Relapse#1: 8 (67) Relapse#2: 1 (8) Relapse>2: 1(8) Not specified: 2 (17)	n.a.	n.a.
(Re)-induction prior to SCT	R-DHAP: 5 (42) R-Benda: 4 (33) R-BendaFlu: 2 (17) R-DHAP/R-Benda: 1 (8)	R-CHOP: 13 (62) R-CHOP/R-DHAP: 8 (38)	n.a.
Disease status at SCT	CR: 6 (50) PR: 5 (42) SD: 1 (8)	CR: 9 (43) PR: 10 (48) Not specified: 2 (9)	CR: 15 (46) PR: 15 (46) SD: 1 (3) Not specified: 2 (6)
Disease state at last follow-up after SCT	CR: 10 (83) PR: 1 (8) Relapse: 1 (8)	CR: 16 (76) PR: 2 (10) Relapse: 2 (10) n/a: 1 (5)	CR: 26 (79) PR: 3 (9) Relapse: 3 (9) n/a: 1 (3)
Stem cell source (BM/PSC)	0/12 (0/100)	0/21 (0/100)	0/33 (0/100)
Donor: mrd/mud	3/9 (25/75)	5/16 (24/76)	8/25 (24/76)
HLA antigen-mismatch	3 (25)	2 (9)	5 (15)
SCT: female → male	2 (17)	4 (19)	6 (18)
CMV: neg → pos	2 (17)	5 (24)	7 (21)
Conditioning therapy	TreoFlu: 11 (92) BuCy: 1 (8)	TreoFlu: 15 (71) BuCy: 6 (29)	TreoFlu: 26 (79) BuCy: 7 (21)
CD34^+^-cell dose per kg bodyweight × 10^6^	5.9 (3.2–12.2)	7.1 (3.9–14.9)	6.8 (3.2–14.9)

*SCT* stem cell transplantation, *CTX* chemotherapy, *BM* bone marrow, *PSC* peripheral stem cells, *R* rituximab, *CR* complete remission, *PR* partial remission, *SD* stable disease

*No patient had a history of high-dose therapy followed by autologous stem cell transplantation prior to inclusion into trials #060 and #074

Both protocols and an amendment of protocol #060 were approved by the ethics committee of each participating center and followed the declaration of Helsinki. Informed consent from each patient was mandatory before inclusion [16].

### Follow-up

Follow-up data were collected 6 months from the participating centers. Database was closed for analysis in autumn 2019. Data analysis was performed as previously described [16].

### Quality-of-life assessment

The intention to collect and analyze Quality-of-life data from all long-term survivors was approved by the ethics committee of the University of Greifswald. The EORTC quality-of-life form QoL-C30 was sent out to all surviving patients with a cover letter [17]. The answers were pooled into the three groups “global health status,” “functional scales,” and “symptom scales” and median, mean, range, and EORTC quality-of-life (QoL) score were calculated for each group (https://www.eortc.be/qol/files/SCManualQLQ-C30.pdf).

### Molecular detection of minimal residual disease and chimerism analyses

Minimal residual disease detection and chimerism analyses have been described comprehensively previously [16]. For MRD-analysis, the translocation t(11;14) was the preferred marker. In patients with *t*(11;14) negative MCL, the clonospecific CDR-III regions were used instead.

## Results

### Relapses

Overall, five patients from both trials (OSHO#60: *N*=1, OSHO#74: *N*=4) experienced a clinical relapse of their MCL between 0.5 and 5.9 years after allogeneic transplantation. Two patients died from complications related to their MCL, one patient successfully underwent salvage therapy and second allogeneic transplantation and is well and alive, and two patients received non-transplant salvage strategies.

### Graft-versus-Host disease

Data about acute Graft-versus-Host disease have been presented in our previous paper. The incidence of chronic GvHD was 15% (ltd. disease *n*=5, ext. disease *n*=1) without dynamic or mortality since 2014.

### Mortality

One patient (#1) died prior to stem cell transplantation from progressive lymphoma.

The mortality after alloSCT was 27% (9/33 patients, OSHO#60: N=4, OSHO#74: N=5) (Table 2). In detail, patients #2 to #10 died between day +8 and 5.9 years after transplantation from relapse (*N*=2), infections (*N*=5), bleeding in conjunction with aspergillosis (*N*=1), and toxicity (*N*=1).

**Table 2:** Mortality

Pat.	Trial	G	Age at SCT (years)	Follow-up after allo-SCT	Causes of death	Remarks
#1	OSHO #060	M	65	n. a.	Progressive disease	Patient did not proceed to allo-SCT
#2	OSHO #060	M	64	Day +8	Infection in aplasia	
#3	OSHO #060	M	61	Day +8	Kidney and lung toxicity IV° plus pneumonia	Conditioning: treosulfan/fludarabine
#4	OSHO #060	M	64	Day +481	Septic cardiomyopathy	
#5	OSHO #074	F	63	Day +15	Bleeding due to Aspergillosis of the CNS	
#6	OSHO #074	M	69	Day +312	Infection	Patient with relapse of MCL_BV_ after allo-SCT had received DLI and developed GvHD IV°. Death from infection in CR of MCL_BV_ and GvHD IV°
#7	OSHO #074	M	59	Day +9	Infection	
#8	OSHO #074	M	59	Day +1009	Infection	Septicaemia with Ps. aeruginosa_MBL_ and E. coli_ESBL_ due to diabetes mellitus related abscess
#9	OSHO #074	M	63	Day +229	Progressive disease	Relapse diagnosed at day +213
#10	OSHO #60	M	60	Day +2168 (5,9 years)	Progressive disease	

*G* gender, *MBL* metallo-beta-lactamase, *ESBL* extended-spectrum beta-lactamase, *MCLBV* blastic variant of mantle cell lymphoma, *DLI* donor lymphocyte infusion

### Progression-free and overall survival

#### All patients

**Fig. 1:**
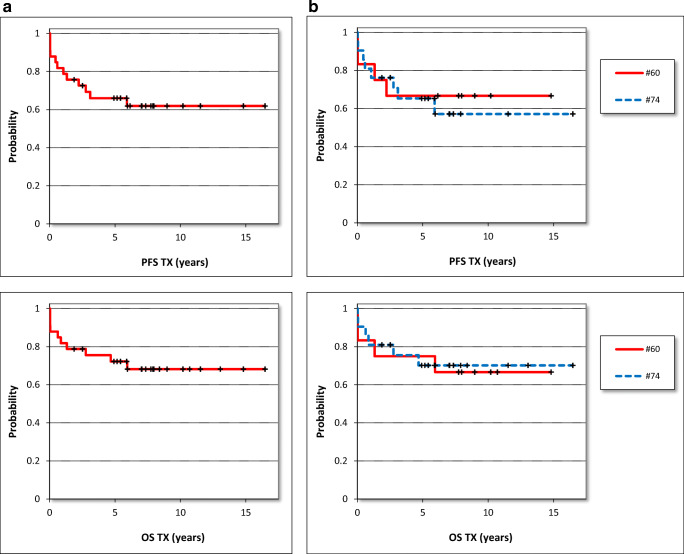

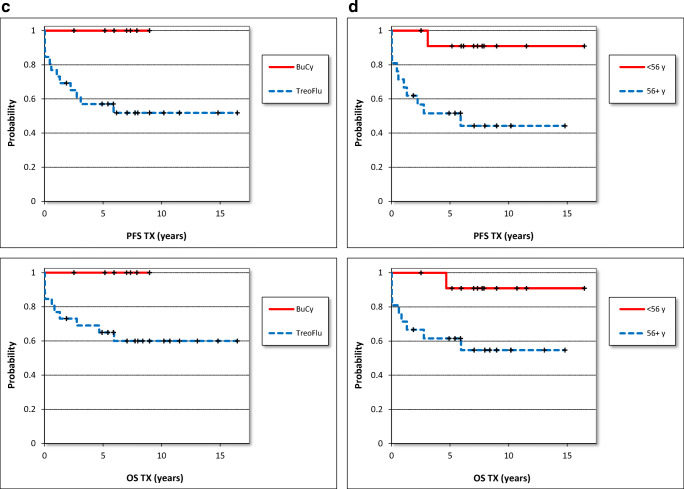
Progression-free survival (PFS) and overall survival (OS) after allogenic stem cell transplantation in years. **a** All patients (*n*=33), **b** salvage (*n*=12, OSHO #060) vs. primary (*n*=21, OSHO #074) SCT, **c** toxicity-reduced (*n*=26, TreoFlu) vs. myeloablative (*n*=7, BuCy) conditioning (PFS: *p*=0.04, log-rank test), **d** patients <56 years (*n*=12) vs. patients ≥56 years (*n*=21) (PFS: *p*=0.01, OS: *p*=0.04, log-rank test)

The progression-free and the overall survival were 62% and 68% after a follow-up of 16.5 years (Table 3). The median progression-free (PFS) and overall survival (OS) for all 33 patients were 5.9 (median, range 0.02–16.5) years after allogeneic stem, cell transplantation. The 50% survival was not reached for both parameters (Table 3, Fig. 1).

**Table 3:** Progression free survival (PFS) and overall survival (OS) for all patients and for subgroups in years

Collective	All patients	OSHO #60	OSHO #74	BuCy conditioning	TreoFlu conditioning	Age <56 years	Age 56+ years
*N*	33	12	21	7	26	12	21
PFS (years)		*n.s.*		*p*=0.04 (log-rank test)		*p*=0.01 (log-rank test)	
Median	5.9	7.9	5.2	7.0	5.2	7.2	2.8
Minimum	0.02	0.02	0.02	2.5	0.02	2.5	0.02
Maximum	16.5	14.8	16.5	9.0	16.5	16.5	14.8
50%	n. r.	n. r.	n. r.	n. r.	n. r.	n. r.	5.9
PFS total	62%/16.5y	67%/14.8y	57%/16.5y	100%/9.0y	52%/16.5	91%/16.5y	44%/14.8y%
OS (years)		*n.s.*		*n.s.*		*p*=0.04 (log-rank test)	
Median	5.9	8.5	5.4	7.0	5.9	7.5	5.4
Minimum	0.02	0.02	0.02	2.5	0.02	2.5	0.02
Maximum	16.5	14.8	16.5	9.0	16.5	16.5	14.8
50%	n. r.	n. r.	n. r.	n. r.	n. r.	n. r.	n. r.
OS total	68%/16.5y	67%/14.8y	70%/16.5y	100%/9.0y	60%/16.5y	91%/16.5y	55%/14.87y

*n. r.* not reached

#### Salvage vs. primary transplantation

For patients after salvage allograft, the PFS was 7.9 (median, range 0.02–14.8) and the OS was 8.5 (median, range 0.02–14.8) years. Patients with de novo MCL had a median PFS of 5.2 (range 0.02–16.5) years and a median OS of 5.4 (range 0.02–16.5) after transplantation. The patients after salvage transplantation had a non-significant trend for a better PFS and OS compared to patients allografted for de novo MCL. In consequence, PFS and OS were comparable after primary and salvage transplantation. Again, 50% DFS and OS were not reached in any group (Table 3, Fig. 1).

#### Myeloablative vs. toxicity-reduced conditioning

When patients after myeloablative conditioning were compared to those after toxicity reduced conditioning, the PFS was significantly better for the BuCy-group: DFS 7.0 (median, range 2.5–9.0) years compared to 5.2 (median, range 0.02–16.5) years (*p*=0.04, log-rank test). The differences were similar for OS with 7.0 (median, range 2.5–7.9) years compared to 5.9 (median, range 0.03–16.5), however, without reaching significance level of 0.05. Fifty percent PFS and OS were not reached. The progression-free and overall-survival after myeloablative conditioning were 100% after a follow-up of 7.9 years, respectively (Table 3, Fig. 1). After toxicity-reduced conditioning, DFS was 52% and OS was 60% after 16.5 years.

#### Patients <56 years vs. patients 56+ years

Younger patients aged below 56 years had a significantly better PFS compared to elderly patients: 7.2 (median, range 2.5–16.5) years vs. 2.8 (median, range 0.02–14.8) years (*p*=0.01, log-rank test). The differences were similar for the overall survival with 7.5 (median, range 2.5–16.5) years compared to 5.4 (median, range 0.02–14.8) years (*p*=0.04, log-rank Test). The 50% PFS was 5.9 years for patients of 56 years and elder (Table 3, Fig. 1).

It should be pointed out that the comparisons of the outcome by age and by conditioning were not pre-planned by the protocol. A multivariate-analysis was not conducted since all patients of ≥56 years received Treosulfan/Fludarabin as conditioning.

### Other parameters

Patient’s gender or familiar vs. unrelated transplantation had no influence on the outcome (data not shown).

### Quality-of-life

Quality-of-life questionnaires were completed and returned by only seven out of 24 living patients (29%). Therefore, this item has only limited value. Analysis was performed according to the EORTC-guide with the exception that answers from the three topics “global health status,” “functional scale,” and “symptom scale” were pooled for analysis under each subheading. The global health score was very good with 69 (maximum: 100), the functional scale was excellent with 99 (maximum: 100), and the symptom score was very good with 13 (maximum: 100) (Table 4).

**Table 4:** Quality-of-life

Parameter	Questions	Interpretation	Median	Mean	Minimum	Maximum	EORTC-score
Global health status	2	1: poor 7: excellent	5.0	5.1	3	7	69
Functional scales	15	1: no problems 4: strong problems	1	1.7	1	4	99
Symptom scales	13	1: no signs 4: strong symptoms	1	1.4	1	3	13

### Molecular detection of minimal residual disease and chimerism analyses

Samples for long-term molecular follow-up after alloSCT were available from only four patients (Table 5). All patients were MRD-negative and had full donor chimerism after a follow-up between 70 and 166 months. Patients B and C were in the first complete remission at their last follow-up 9.0 and 14.8 years after allogeneic transplantation, respectively. Patient A relapsed 2.2 years after alloSCT and was further treated with non-transplant protocols. He refused a re-transplantation and has died 5.0 years after transplantation from progredient lymphoma. Patient D had relapsed 5.9 years after transplantation, was treated with chemotherapy, and again transplanted from an alternative donor. At his last follow-up, 13.0 years after the first transplantation, he was well and alive in complete remission.

**Table 5:** Molecular follow-up

Patient	Disease status at last follow-up	MRD status at last molecular follow-up	Months after the first alloSCT at last molecular follow-up	Donor chimerism (%) at last molecular Follow-up	Months after the first alloSCT at last chimerism follow-up
A)	4th CR	MRD neg.	70	99	70
B)	2nd CR	MRD neg.	166	100	166
C)	2nd CR	MRD neg.	84	100	108
D)	2nd CR	MRD neg.	126	100	126

## Discussion

The updated results from the prospective OSHO-trials #060 and #074 show the excellent long-term outcome and the curative potential of allogeneic stem cell transplantation in the treatment of mantle cell lymphoma. Data for progression-free and overall survival of 72% and 62% after 16.5 years compare favorable to those from the Seattle group, the sole publication with prospective data beside the OSHO-trials [13]. However, long-term data after nonmyeloablative conditioning did not show a clear survival plateau [14].

The presented analysis of the OSHO trials revealed a better outcome of younger patients compared to elderly. This does not surprise and has been described by other investigators retrospectively [10]. Furthermore, a myeloablative conditioning was associated with an improved outcome in the present investigation. However, since younger patients received the more intensive conditioning and the data do not allow a multivariate analysis, it cannot be clarified if younger age or the choice of conditioning is more important for the outcome of patients. Cook et al. described a 5-year OS and PFS of 37% and 14% for patients with relapsed and refractory mantle cell lymphoma. Le Gouill et al. published an OS and EFS of 55% and 53% after RIC conditioning in a similar collective [12]. The data from the OSHO #060 trial compare favorably to data from both papers; however, it must be mentioned that 34% and 70% of patients in the data from Cook and Le Gouill had undergone a preceding high dose therapy followed by autologous stem cell reinfusion comparing to no patients in the OSHO #060 trial.

There are limited data clarifying the role of myeloablative vs. non-myeloablative conditioning for mantle cell lymphoma. The results of the OSHO trials indicate that survival is better after myeloablative conditioning compared to RIC; however, this might be an effect of patient’s age. A retrospective comparison of MAC vs. RIC by Hamadani et al. for patients suffering from chemotherapy-refractory MCL revealed no differences between both approaches [18]. In general, the choice of a myeloablative regimen in MCL is limited by the fact that most patients are elderly and many patients have a history of intensive pre-treatment.

The importance of MRD-negativity after stem cell transplantation has been addressed by investigators in the allogeneic and the autologous setting [4, 5, 11]. The relevance of MRD-negativity after alloSCT for MCL was already described in our previous publication. [16]. Unfortunately, only samples from four patients were available for this long-term follow-up analysis. However, these limited data show that permanent long-term donor chimerism and MRD negativity can be achieved by allogeneic stem cell transplantation. However, the data also show that relapse of MCL can occur despite full long-term chimerism and long-term MRD-negativity.

Data about the quality-of-life after allogeneic SCT for MCL are lacking in the literature. The presented approach to collect the data was of limited success since the response rate was only 29%. Therefore, results were highly prone to bias. However, the quality-of-life reported by the 29% responders from long-term survivors was good to very good.

The position of allogeneic stem cell transplantation in therapeutic algorithm of mantle cell lymphoma has not been defined so far. Data from a recent CAR-T-cell trial are promising; however, a direct comparison to allogeneic SCT is lacking [15]. High-dose therapy followed by autologous stem cell reinfusion is the accepted standard for consolidation of the first remission even though this procedure does not lead to a survival plateau [3, 8, 19]. Long-term results of HDT + autoSCT can be improved convincingly by maintenance therapy afterwards [6, 7]. Allogeneic transplantation is a standard of care for patients with MCL in higher remissions since other promising therapies with curative potential are lacking and the data from CAR-T-cell therapy need further maturing [15]. Limitations of the presented data are the low number of patients and the partial lack of the prospective risk classification of the patients by the MIPI, since this index was introduced when both trials were already running [20].

In conclusion, alloSCT is a potentially curative therapy for patients suffering from MCL in the first or higher remission with a higher rate of early morbidity and mortality but a better long-term outcome than HDT + autoSCT. A prospective comparision with HDT + autoSCT with inclusion of a maintenance strategy is necessary. Allogeneic stem cell transplantation should be offered to all feasible patients in second remission and this valuable therapeutic option should be discussed with younger patients suffering from high-risk de novo MCL.
